# Characterisation of anthracycline cardiotoxicity in long-term childhood cancer survivors using conventional and novel CMR techniques: probing the pathology

**DOI:** 10.1186/1532-429X-17-S1-P260

**Published:** 2015-02-03

**Authors:** Sadia Quyam, Jennifer Steeden, Vivek Muthurangu, Tanzina Chowdhury, Marina Hughes

**Affiliations:** Great Ormond Street Hospital for Children NHS Foundation Trust, London, UK; University College London, London, UK

## Background

Late cardiotoxicity is a known complication of anthracycline therapy. CMR is the gold standard for assessment of cardiac function, with demonstrated utility in the detection of subtle abnormalities of myocardial structure and motion. Using conventional and novel CMR techniques, we re-examined a cohort of late cancer survivors, previously studied with echocardiography in childhood. We compared them concurrently to healthy controls, aiming to characterise myocardial abnormalities in the context of longitudinal functional data.

## Methods

30 patients exposed to anthracyclines in childhood and 19 age and sex-matched controls underwent conventional functional CMR, T1 mapping, Tissue Phase Mapping (TPM) and detailed echocardiography. Myocardial T1 maps were obtained using a Modified Look-Locker Inversion Recovery (MOLLI) sequence. Native T1 maps were obtained in controls, and patients were given 0.15mmol/kg gadolinium for extracellular volume (ECV) quantification. Respiratory self-gated TPM was performed, using a rotating golden-angle spiral acquisition with retrospective cardiac gating. Both were acquired in the left ventricular (LV) mid-short axis.

## Results

The patients' mean age was 31 ±5 years and they were studied a median of of 25 (range 16-32) years following a median cumulative anthracycline dose of 220 (range 90-370) mg/m^2^ for either leukaemia or Wilms tumour.

Mid-term fractional shortening (FS), 8 years following therapy had a mean value 33.1 ±5.1%. Current CMR-derived LVEF was normal, although reduced compared to controls (61% ±6 vs. 65% ±5, p <0.01) and correlated negatively with cumulative anthracycline dose (R^2^ 0.26, p <0.01) and positively with mid-term FS (R^2^ 0.27, p <0.03).

Native myocardial T1 was not significantly different, comparing patients and controls (967 ±37 vs. 960 ±37, p 0.53). Mean ECV was 0.26 ±0.04 and was higher in patients receiving cumulative doses of >300mg/m^2^ (0.25 ±0.02 vs. 0.28 ±0.05, p = 0.02).

TPM-derived LV radial, longitudinal and tangential systolic velocities, and longitudinal E/A ratio were not significantly reduced compared to controls.

## Conclusions

This is the first detailed assessment of myocardial function using novel CMR techniques in survivors, 25 years following childhood anthracycline therapy. Mild, asymptomatic, dose-related reduction in LVEF is seen, with no associated change in native T1 values. Increased ECV is seen only at cumulative doses >300mg/m^2^. Previous studies have shown significantly increased ECV, a median of 8 years post therapy. Our finding, associated with detailed myocardial motion analysis, provides further insight into the mechanisms of long-term remodeling in this specific cohort.

## Funding

Dr Quyam was funded by a start-up grant from CHILDREN with CANCER UK

Registered charity no. 298405.

**Table 1:** Control and Patient group characteristics and cardiovascular MR data

VARIABLE	CONTROL (n = 19)	PATIENTS (n = 30)	p value
Female n (%)	10 (53)	17 (57)	0.86
Current Age (years)	32 (26-39)	32 (23-42)	0.96
Height (cm)	171 ±6.3	169 ±9.2	0.3
Weight (kg)	67 ±8	75 ±21	0.1
NYHA I / II	19 (100) / 0 (0)	28 (93) / 2 (7)	0.44
Systolic BP (mmHg)	120 ±10	123 ±15	0.46
Diastolic BP (mmHg)	73 ±7	75 ±11	0.6
AML n (%)		2 (7)	
ALL n (%)		23 (77)	
Wilms n (%)		5 (17)	
Age at diagnosis (years)		4.6 (1-15)	
Time since chemo (years)		25 (16-32)	
Anth Dose (mg/m2)		220 (90-370)	
Radiotherapy n (%)		24 (80)	
BMT n (%)		7 (23)	
CARDIOVASCULAR MR DATA			
LVEDV index (ml/m2)	72 ±8	73 ±12	0.92
LVESV index (ml/m2)	25 ±6	30 ±8	*0.05
LV EF (%)	65 ±5	61 ±6	*0.01
LV mass index (g/m2)	60 ±10	58 ±11	0.53
RVEDV index (ml/m2)	76 ±8	69 ±12	0.10
RVESV index (ml/m2)	29 ±6	28 ±14	0.68
RV EF (%)	62 ±4	63 ±5	0.39
MAPSE (cm)	15 ±1.1	13 ±2.6	*0.02
Left Atrial Area (cm2)	20 ±2.3	21 ±3.1	0.47
Native myocardial T1	960 ±37	967 ±37	0.53
Native blood pool T1	1484 ±76	1463±118	0.50
Extracellular volume fraction		0.26 ±0.04	
Focal LGE		2 (7)	

Values expressed mean mean ±SD.

LVEDV = left ventricular end diastolic volume; LVESV = left ventricular end systolic volume; LVEF = left ventricular ejection fraction; RVEDV = right ventricular end diastolic volume; RVESV = right ventricular end systolic volume; RVEF = right ventricular ejection fraction; MAPSE = mitral annular plane systolic excursion; LGE = Late gadolinium enhancement.

**Table 2:** Data comparing high and low cumulative anthracycline dose.

CARDIOVASCULAR MR DATA	Low dose <300mg/m2 (n =22)	High dose >300mg/m2 (n = 8)	p Value
LVEDV index (ml/m2)	71 ±11	80 ±14	0.1
LVESV index (ml/m2)	28 ±8	36 ±6	*0.03
LV EF (%)	65 ±5	54 ±6	*0.01
LV Mass index (g/m2)	58 ±11	59 ±9	0.94
RVEDV index (ml/m2)	70 ±10	69 ±17	0.87
RVESV index (ml/m2)	28 ±15	26 ±7	0.77
RV EF (%)	65 ±5	62 ±2	0.46
MAPSE (cm)	13 ±2.1	13 ±4.1	0.72
Left Atrial Area (cm2)	21 ±3	21 ±4	0.94
Native T1	960 ±39	992 ±24	*0.05
Native blood pool T1	1464 ±79	1527±38	*0.05
Extracellular volume fraction	0.25 ±0.02	0.28 ±0.05	*0.02
LGE	1 (14)	1 (5)	

Values expressed mean mean ±SD

LVEDV = left ventricular end diastolic volume; LVESV = left ventricular end systolic volume; LVEF = left ventricular ejection fraction; RVEDV = right ventricular end diastolic volume; RVESV = right ventricular end systolic volume; RVEF = right ventricular ejection fraction; MAPSE = mitral annular plane systolic excursion; LGE = Late gadolinium enhancement

